# Re-engineering the laboratory

**DOI:** 10.1155/S1463924696000120

**Published:** 1996

**Authors:** Frank Zenie

**Affiliations:** Zymark Corporatiom Zymark Center Hopkinton MA 01748 USA


Journal of Automatic Chemistry, Vol. 18, No. 4 (July–August 1996), pp. 135–141

### Re-engineering the laboratory

**Frank Zenie**
*Zymark Corporatiom, Zymark Center, Hopkinton, MA 01748*

*This paper explains why, in today's rapidly changing environment, it is essential for organizations to re-engineer. The main drivers behind business re-engineering are informed customers and information technology. The author explains the changes needed in management behaviour and performance and then describes, step by step, how to re-engineer laboratories. Finally, the importance of partnerships is stressed and explained by examples.*

'If it ain't broke, don't fix it!' We hear this all the time. While it is intended to be prudent advice, it is based on a flawed premise. It assumes that competitors, customers and the economy will remain constant. Yet, given our rapidly changing world, this cautious approach is really a high risk philosophy. We either embrace change or become followers of the new leaders.

Our organizations are changing more rapidly than ever before—and the consequences range from success and prosperity—to fragile survival—to economic failure. Re-engineering is a process through which we can initiate and direct change rather than react to changes created by others.

Re-engineering business is not new, yet it is timely to meet today's challenges: 'In today's environment, nothing is constant or predictable—not market growth, customer demand, product life cycles, the rate of technological change, or the nature of competition... It is not products but the processes that create products that bring companies long term success. Good products don't make winners; winners make good products [1]'.

Two primary forces driving these changes include:

(1) *Informed customers*—Informed customers demand more value and, thereby, stimulate competition. Today's individual consumers and business purchasers are well educated and demand higher quality, service and value. Providers of products and services, therefore, must re-earn customers' business by providing innovative products and greater value.
(2) *Information technology* (IT)—IT is a driving force and an enabling technology facilitating innovation and change. IT enables organizations to shift from inefficient hierarchies to efficient teams working on high leverage opportunities. Ever increasing globalization, for example, is a strategic opportunity made possible only through advances in IT.

These driving forces are irreversible and relentless, that is, they will never return to the past. Regulation, on the other hand, is often a political and even emotional driving force. We have seen many examples where the desired benefits of regulation are better attained through education and economic incentives.

Science cannot separate itself from business and economics. Science creates the technology which drives our most robust businesses. As competitive, consumer and regulatory pressures force business to improve quality and productivity, science must lead, or at least enable, the bold steps ahead.

Not too many years ago when a company hired a new scientist, that scientist could purchase his or her favourite instrumentation, computers and software. This is no longer true. With today's need for information sharing, technology transfer and regulatory compliance, companies must harmonize and standardize their use of technology.

### Profit leverage—beyond cost reduction

Near term business challenges trigger restructuring programmes to reduce costs and maintain profitability. Longer term, focus will shift to re-engineering business processes to make organizations more productive by creating greater customer value.

To begin, we need new definitions and measurements of costs. In the past, we turned to the accountants to measure the 'cost' of our decisions. ROI (Return on Investment) became the commonly accepted measure to evaluate investment opportunities. Yet, ROI only recognizes accounting costs.

*Accounting costs* are tangible and relatively easy to measure and control—much of today's downsizing, for example, is focused on reducing accounting costs. *Opportunity costs*, on the other hand, include intangible and indirect costs including the lost income from missed business opportunities. Only time will tell if the opportunity costs of today's decisions exceed the short term accounting savings. Both accounting costs and opportunity costs are real! Just because intangible costs may never, or not until some time in the future, be recognized through accounting, does not diminish their strategic impact. More often than not, the highest business leverage relates to opportunity costs of missed business opportunities.

Effective use of time and resources are critical, therefore, faster time to market becomes a strategic initiative to maximize the return on successful projects. While more difficult to measure, 'failing fast' saves opportunity costs and permits reallocating valuable resources from a failed project onto a new, more promising one. Sooner is generally better than later.

### Accelerating change in chemistry

Modern chemistry began with exciting discoveries during and following the Second World War. Over the past 10 to 20 years, we have been digesting and applying these

0142-0453/96 $12.00 © 1996 Taylor & Francis Ltd.

new technologies. Nowhere is this more evident than in drug discovery—driven by the huge economic reward for powerful new therapies.

During the 1980s, scientists recognized the analytical power of computers and many extended this power to computerized molecular development—rational drug design. Molecular and therapeutic knowledge proved insufficient to meet this challenge and few, if any, drugs were discovered through purely rational processes.

Entering the 1990s, pharmaceutical and biotechnology scientists and executives recognized that they had to return to the drug discovery techniques that created most modern drugs and apply new technology to make these techniques far more productive.

Using 1990 for reference, typical drug screening projects completed 10 000 to 20 000 assays per year against one target. Large pharmaceutical companies with large compound libraries (100 000 to 200 000 compounds) were well stocked and their organic synthesis staff were adding 50 to 100 compounds per year per scientist.

Today, High Throughput Screening (HTS) can perform 500 000 to 1 000 000 assays per year, often with multiple targets. Automated organic synthesis (combinatorial and parallel synthesis techniques) can produce thousands of compounds per week. These two revolutions, combined with powerful data management innovations, make today's drug discovery efforts three to five orders of magnitude more productive than those at the start of this decade.

### Technology—enabling re-engineering

The goal should now be to increase productivity—that is create more economic value for each unit of cost. This is different from cost reduction. Wherever possible, people and automation should be teamed for productivity. Re-engineering involves a systematic assessment of cross-functional, work flow processes which stimulate creative approaches to streamline the processes, introduce innovation and to eliminate unnecessary steps and hand-offs. New technologies enable more creative re-engineering solutions.

The dramatic productivity gains in drug discovery, for example, were enabled by technology advances (see table 1). New technology is increasing drug discovery productivity by several orders of magnitude compared to a few years ago. With more leads and more powerful lead optimization, more and better drug candidates will require development support—further challenging development resources. Again, technology is enabling development to meet this challenge, for example, bioanalytical support of clinical trials (see table 2). Independently, each of the technologies in table 2 contributes incremental productivity. When combined in a new process, however, far greater productivity improvements become possible.

Figure 1 illustrates the relative costs for gaining each increment of productivity benefit. When driven by technology or people reduction, we often push into the region of diminishing leverage. In laboratory automation,

*Table 1. Enabling technologies for drug discovery.*

| Technology | Benefit |
|---|---|
| Innovative screening assays and automated high throughput screening (HTS) | One to two orders of magnitude increase in screening capability. |
| Automated organic synthesis and combinatorial chemistry | Two to three orders of magnitude increase in compounds for HTS. More diverse compound arrays. |
| Automated compound preparation and distribution | Capacity to provide large numbers of compounds for HTS. Create and access compound data base. |
| Computers and data management | Tracking and analysing discovery data. Global integration of drug discovery programmes. |
| Automated structural characterization | Timely availability of improved compound knowledge for screening and lead optimization. |

*Table 2. Enabling technologies for bioanalytical analysis.*

| Technology | Benefit |
|---|---|
| Overnight package delivery | Timely delivery of supplies to the clinic and timely delivery of patient samples to the laboratory. |
| Communications and information systems | Real time information sharing. |
| LC/MS/MS | Very fast (2–5 minutes per sample), high resolution analysis of biological samples. Rapid methods development. |
| High throughput extraction and concentration | Dramatic increase in sample preparation throughput, improved analytical data and less solvent consumption for bioanalytical methods. Rapid methods development and optimization. |

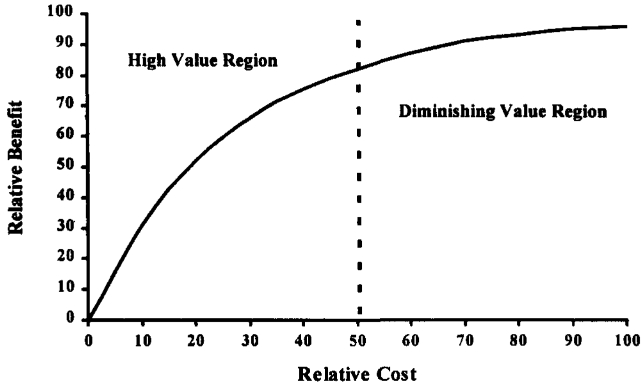

*Figure 1. Costs for gaining each increment of productivity benefit.*

this translates into increasing complexity, higher cost and decreasing reliability. The most successful laboratory automation projects focus on the 'high value region'. When a little automation is good, it does not mean a lot of automation is better.

### Management role

Today, improving productivity is management's primary mission. In the early phase of the industrial revolution, managers adopted the military command and control model—managers controlled workers who performed physical tasks. Over time, physical work was transferred to machines and workers gained more education to become 'knowledge workers'. Following this, expert knowledge workers became managers to manage specialized functions. Hierarchical organizations evolved to control work and facilitate the flow of information. Until recently, managers focused on specialized tasks rather than cross-functional processes which produced value. It is not surprising that serving customers got lost.

The industrial revolution is now the information revolution. Leadership is no longer defined by hierarchical position. Productivity and value must be viewed within a work flow process, not limited by functional boundaries. Leaders bring knowledge, commitment, and shared values and purpose. 'Managers stop acting like supervisors and behave more like coaches. Workers focus more on the customers' needs and less on their bosses' [2].

### Management survey

In order to gain further insights into management's perspective on laboratory automation, I surveyed a small group of senior managers for their observations. The managers were directors, senior directors and divisional vice presidents. The survey group was limited to individuals with direct experience with laboratory automation in their areas and a management vision relating to strategic impact. Twenty managers responded, from four functional areas: drug discovery; analytical R&D; bioanalytical R&D; and quality assurance/quality control. The survey explored automation objectives and desired improvements to make automation more valuable. Survey participants rated each topic at one of three levels: very important, important, or relatively unimportant. Since the survey group was small, the summary results in table 3 are shown as relative responses.

Table 3 shows that laboratory automation's primary objective is to increase laboratory capacity for higher work loads and increase productivity of limited staff. Faster sample turnaround also was a shared priority. As expected, R&D managers see laboratory automation reducing new product time to market. Quality control managers, on the other hand, focus on reducing manufacturing cycle time and maintaining regulatory compliance. Surprisingly, methods and technology transfer was not seen as a strong objective for laboratory automation (see table 4). Managers from all areas stressed flexibility to meet rapidly changing needs. Most likely, R&D managers emphasize flexibility in order to adapt to new projects and new technologies, while QC managers need flexibility to automate a growing number of methods needed for broader product lines and smaller manufacturing batches required by rapidly changing market demand.

### Laboratory re-engineering—a systematic process

### *Getting ready*

Prior to solving any problem, we must gain agreement that there is a problem and that it is worth solving. Only then, can we begin the solution process. Re-engineering is a solutions process that demands intellectual contributions and commitment from the people involved. Without this, don't start.

Effective laboratory re-engineering requires systematic analysis and creative solution development. Defining process boundaries is critical; if too narrow, the solution

*Table 3. Management survey.*

| Automation objectives | All | R&D | QA/QC |
|---|---|---|---|
| Increased capacity for higher work loads | High + | High + | High + |
| Increased productivity—lower cost or staffing limitations | High + | High + | High + |
| Financial returns—ROI | Moderate | Moderate | Moderate |
| Development—reduce time to market | Moderate | High | Low |
| Faster sample turnaround | High | High | High |
| Improved precision documentation and audit trails (validation) | Moderate | Moderate | High |
| Improved data management | Moderate | Moderate | Moderate |
| Regulatory compliance | Moderate | Low | Moderate |
| Facilitate methods transfer—analytical to QC, multisite, outsourcing | Moderate | Low | Moderate |
| Global unification of lab methods | Low | Low | Low |
| Reduce the use of contract labs | Low | Low | Low |
| Reduce manufacturing cycle time | Moderate | Low | High |
| Staff motivation and job enrichment | Moderate | Moderate | Moderate |
| Staff safety | Moderate | Moderate | Moderate |
| Better space utilization | Low | Low | Low |

*Table 4. Improvements which would make laboratory automation more valuable.*

| | All | R&D | QA/QC |
|---|---|---|---|
| Improved reliability | High | High | High |
| Ease of use and faster implementation | High | High | High |
| Flexibility—reconfigure for new applications | High + | High + | High + |
| More powerful connectivity | Moderate | Moderate | Moderate |
| Improved methods development capability | Moderate | Moderate | Moderate |
| Lower cost | Moderate | Moderate | Moderate |

may have limited impact and, if too broad, the solution may be too difficult to implement. Fortunately, people working in the area of interest typically understand the issues and problems. Re-engineering leaders, in most cases, serve as facilitators extracting and analysing readily available information. The re-engineering process follows four phases: situation analysis; identify solutions and opportunities; select high leverage solutions; and implementation—plan and execution.

### *Situation analysis*

Process mapping graphically highlights work flow, hands-off and redundant activities. The generic process map, shown in figure 2, illustrates two critical pharmaceutical industry processes and their organizational interactions. First, is the manufacturing/quality-control process focused on the flow of raw materials through finished products released for distribution. The second, illustrates the analytical methods development process from early development to NDA filing and to QC for global, multiplant use. Beginning with this generic process map. It is easy to modify it to represent each organization specific work flow.

### *Process trends assessment*

The next step in situation analysis requires analysis of process trends and complexity. Highly complex processes tend to become inefficient and unpredictable. Table 5 is an approach to identify process trends and complexity in a pharmaceutical manufacturing and QC situation. Using the definitions in table 5, an individual organization can graphically highlight its process trends and complexity under current conditions and projected at some appropriate time in the future. Table 6 illustrates the following process trends:

(1) The number of different products is growing due to new products and the transfer of some existing products to this manufacturing site.
(2) Even though the number of products is growing, the manufacturing plants are specializing in specific dosage forms.
(3) The company wants to be more responsive to market demand, yet it wants lower inventories and faster cycle time. The company, therefore, will produce smaller batches in response to current market demand.
(4) The fundamental manufacturing process will not change greatly.
(5) Analytical methods are becoming more complex to meet regulatory demands.
(6) New product introductions will increase over historical levels.

### *Product leverage assessment*

Just as process trends help identify re-engineering opportunities, product trends offer additional insights (see figure 3). The product leverage assessment in figure 3 indicates the re-engineering insights in Table 7.

### *Identify solutions/opportunities*

This phase of re-engineering is illustrated in table 8.

### *Select high leverage solutions*

The symptom–cause–solutions process identifies many potential solutions. The highest leverage solutions should

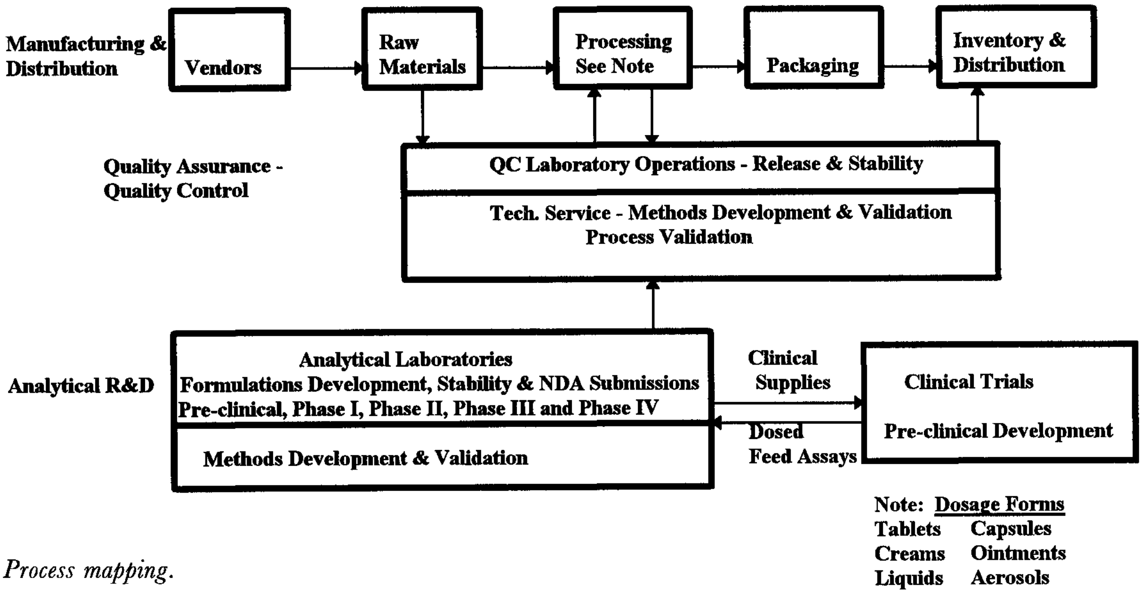

*Figure 2. Process mapping.*

*Table 5.*

| Process complexity | Low | Moderate | Substantial | High |
|---|---|---|---|---|
| Number of different products produced | One or two related | Several related | Several related Some unrelated | Many and unrelated |
| Dosage form varieties | Single | Several related | Several, some unrelated | Multiple and diverse |
| Planning complexity—demand variations—peaks and valleys | Uniform and predictable demand | Variable, but with planning lead time | Variable, with little lead time | Unpredictable reactive planning |
| Complexity of individual process steps—yield | Simple and stable | Somewhat complex, but robust | Complex—not robust | Complex—not yet robust |
| Analytical complexity | Simple and fast, can be performed by operator | Reliable, but requires trained technician | Technique dependent, requires skilled analyst | Utilize new techniques, requires methods development support |
| New product introductions | None | Occasional and related to current products | Several per year, but related to current products | Several per year requiring new processes and analyses. |

*Table 6.*

| Process complexity | Low | Moderate | Substantial | High |
|---|---|---|---|---|
| Number of different products produced | | ○ ——— | ——→ ● | |
| Dosage form varieties | | | ○● | |
| Planning complexity—demand variations—peaks and valleys | | ○ ——— | ——— | ——→ ● |
| Complexity of individual process steps—yield | | ○ ——— | ——→ ● | |
| Analytical complexity | | ○ ——— | ——→ ● | |
| New product introductions | | ○ ——— | → ● | |

○ Today
● Projected 3 years.

be selected for implementation from these. Figure 4 is a useful model for selecting high leverage solutions. Not only do the highest leverage solutions deliver high value, they also establish credibility for the process and the participants.

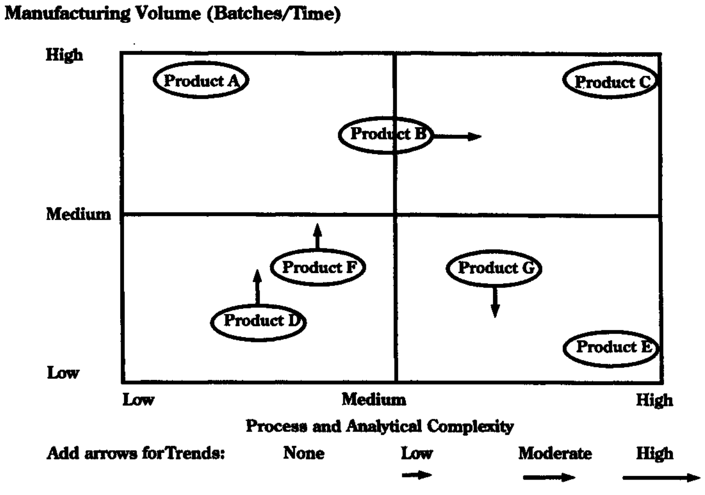

*Figure 3. Manufacturing volume (batches/time).*

*Implementation*

Shortcuts often lead to wasted money, time and resources. Figure 5 illustrates Zymark's solutions implementation process for laboratory automation projects. It emphasizes planning, management involvement, training and long-term support. Earlier technology's role for enabling re-engineering was highlighted. For enduring success, technology must be transferred to the operational staff.

### Partnering for productivity

The business world is changing and the traditional buyer/seller relationship no longer meets many of today's challenges. Organizations are focusing their limited resources on core competencies, and yet, non-core areas are more complex than ever before. Innovative organizations will partner with specialized organizations to gain access to the critical mass of knowledge and skilled resources needed to address these issues.

Two examples illustrate the partnering trend. Biotech companies are more and more becoming the research

*Table 7. Re-engineering insights.*

| Product | Re-engineering leverage issues |
|---|---|
| Product A | High volume, low complexity. Probably very profitable. Re-engineering might make it even more productive. |
| Product B | Relatively high volume and moderate, but increasing complexity. Good candidate for re-engineering. |
| Product C | High volume, high complexity. Good candidate for re-engineering. |
| Product D | Low, but increasing, volume and low complexity. Re-engineering might have an effect. |
| Product E | Low volume, high complexity. Consider dropping product unless high profit or strategically important. Less frequent, larger batches might help productivity. |
| Product F | Moderate volume and complexity. Might benefit from re-engineering processes shared with other products. |
| Product G | Similar to F, but with decreasing volume. Probably not re-engineering candidate. |

*Table 8.*

| Symptom | Possible causes | Potential solutions |
|---|---|---|
| *Schedule related* | Late raw materials | Improve vendor delivery performance |
| Delays | Inadequate capacity—manufacturing or laboratory | Improve process reliability |
| Late shipments | Process unreliability—low or unpredictable yield | Reduce cycle time |
| | Poor work flow—too many hands-offs | Staffing—add, train, overtime, temps |
| | Inadequate or uptrained staff | Add equipment, preventive maintenance |
| | Insufficient or non-working equipment | Improved planning—decrease batch size |
| | Priority interruptions | Improve work flow and/or laboratory methods |
| | Laboratory backlog—slow turnaround | Introduce efficient, automated methods—multishift operation and at-line testing |
| | Unreliable methods—reruns | Reduce unnecessary handling, transfers and delays |
| *Cost related* | | |
| Low yield | Inefficient process | Improve process efficiency, increase batch size |
| Scrap and reruns | Inadequate capacity—manufacturing or laboratory | Vendor certification |
| Inventory cost—long cycle time | Process unreliability—low or unpredictable yield | Introduce in-process testing to reduce scrap |
| | Raw material quality | Introduce efficient, automated methods—multishift operation—more timely information at lower cost |
| | Human errors | |
| | Small batch size—wide product mix | |
| | Inadequate process control information | Introduce at-line testing—faster release |
| *Quality related* | | |
| Reruns | Process control | In-process testing |
| Scrap | Raw materials or process materials | Introduce efficient, automated methods—more robust analysis and documentation |
| Compliance—reruns and investigations | Unreliable processes or methods | |
| | Inadequate documentation | |

partners for large, multi-national pharmaceutical companies. The emerging biotech industry attracts entrepreneurial scientists and executives, stimulates innovation and retains the flexibility of small organizations. The large companies have the know-how and investment base for

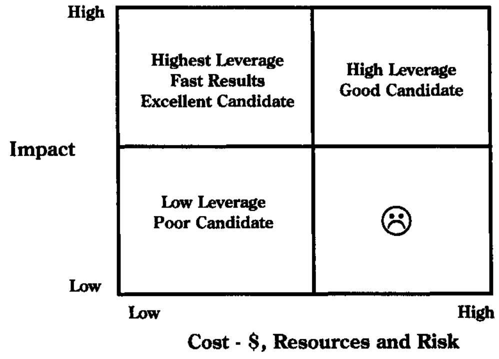

*Figure 4. Model for selecting high leverage solutions.*

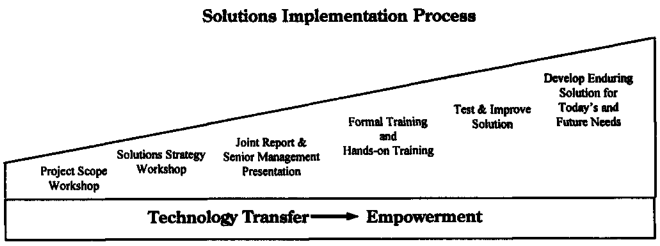

*Figure 5. Solutions implementation process.*

clinical trials, product development, manufacturing and distribution. CROs (contract research organizations) are also becoming more complete partners for the design and implementation of clinical trials.

Organizations cannot simply decide to partner. They must develop relationships, share information and trust each other. The path to partnering is: transaction → solution → partnering.

*Solutions—products and value-added services*

A product, no matter how technology rich, is only part of a solution. Today's solutions are product/service packages. Four examples highlight Zymark's solutions strategy in laboratory automation:

(1) *ISLAR*—A strong educational and networking forum for sharing experiences and knowledge of laboratory automation. In addition, ISLAR is becoming a vehicle to stimulate technology transfer within customer organizations.
(2) *AiS automation and integration services) program*—A training programme which enables customer automation specialists to access and apply Zymark technology at the component and module level.
(3) *Validation consulting*—Many pharmaceutical development and manufacturing processes must be validated under current good manufacturing practices (cGMP) required by the FDA in the USA. As laboratory automation became widely used, pharmaceutical companies were faced with two problems. First, to determine what is required to validate laboratory automation equipment and second, providing the resources to perform the validation. Clearly, it was more efficient for the manufacturers of laboratory automation to apply validation principles to their technology, develop compliant validation protocols and offer professional services to perform the validation. This solution combines both knowledge and resources.
(4) *Automated QC release testing*—A tailored package of automated workstations, methods optimization, training, validation and data management for the QC release of pharmaceuticals. This technology enables the additional step of transferring release testing from the central control laboratory to at-line testing.

*Partnering—interdependent relations*

At Zymark, we had the opportunity to participate in research conducted by Huthwaite, Inc., a leading research-based sales and management consulting firm. Their research into partnering was published in a book *Getting Partnering Right—How Market Leaders are Creating Long-Term Competitive Advantage* published by McGraw-Hill. Huthwaite defines partnering as the ways that corporations are working with each other to create value. Their research identifies three ingredients for successful partnering: vision; impact; and intimacy (see figure 6).

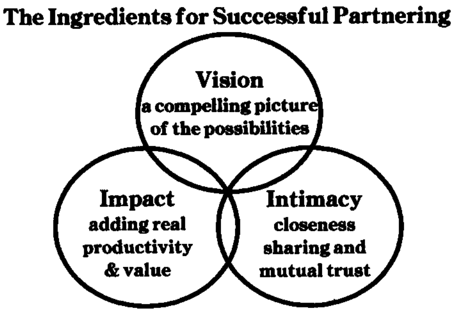

*Figure 6. Successful partnerships.*

Partnering clearly steps beyond the traditional transaction-based relationships. Partnering is not discounting, but includes a sharing of the savings achieved by the closer relationship. Partnering is not appropriate for all organizations or for all aspects of their business. A partnering relationship opens new alternatives to collaborate for productivity, for example:

(1) Re-engineering consulting for cross functional processes—Collaborating with analytical R&D and quality control to introduce automation earlier in the development process. Providing improved methods development tools and support to lower the entry threshold for laboratory automation. Making laboratory automation part of the NDA process for more effective NDA filings and to facilitate later stage development and QC needs for larger sample loads. Collaborating with manufacturing and QC to streamline the in-process and release testing to shorten the manufacturing cycle and lower testing cost under full cGMP compliance.
(2) Creating an alternative to outsourcing and contract laboratories; such as 'in-sourcing', a virtual contract laboratory providing the benefits of outsourcing with increased management control and lower, but still variable, cost.
(3) Joint development of new automation technology for organic synthesis—flexible and concurrent with chemistry developments.

### References

1. Hammer, M. and Champy, J., *Re-engineering the Corporation* (Harper Collins Publishers, New York, 1993).
2. *Ibid.*, p. 65.

